# The impact of intraoperative neuromonitoring combined with evidence-based nursing on vocal cord function, emotional state, pain, and quality of Life in patients after total thyroidectomy for thyroid cancer: a comprehensive study

**DOI:** 10.3389/fonc.2025.1611729

**Published:** 2025-07-15

**Authors:** Dandan Chen, Kun Shang

**Affiliations:** Department of Anesthesiology and Perioperative Medicine, Henan Provincial People’s Hospital, People’s Hospital of Zhengzhou University, Zhengzhou, China

**Keywords:** thyroidectomy, intraoperative neuromonitoring, evidence-based nursing, vocal cord recovery, thyroid cancer

## Abstract

**Objective:**

To evaluate the impact of intraoperative neuromonitoring (IONM) combined with evidence-based nursing on vocal function, emotional status, pain levels, and quality of life (QoL) in patients undergoing total thyroidectomy for thyroid cancer.

**Methods:**

A single-center randomized controlled trial was conducted. The intervention group received IONM with evidence-based nursing, while the control group underwent IONM with conventional nursing. Outcomes were assessed using the Hospital Anxiety and Depression Scale (HADS), Numerical Rating Scale (NRS) for pain, EORTC QLQ-C30 for 1-month postoperative QoL, and Voice Handicap Index simplified Chinese version (VHI-10) combined with laryngoscopy for vocal recovery and complications.

**Results:**

Compared to controls, the intervention group exhibited significantly lower postoperative VHI-10 scores (5 (2, 8) vs 7 (4, 11), *P*<0.001), reduced HADS anxiety scores (5 (2, 8) vs 10 (4, 12), *P*<0.001), and lower 24-hour NRS pain scores (3 (1, 4) vs 4 (2.75, 4.25), *P*<0.001). The intervention group also demonstrated marked improvements in QLQ-C30 global health status (83 (73.75, 86) vs 77 (72.75, 80), *P*=0.001), shorter operative duration (92.467 ± 16.916 vs 107.93 ± 24.26 min, *P*<0.001), reduced intraoperative blood loss (16.5 (9.75, 24) vs 23.5 (11.75, 32) mL, *P*=0.005), and lower postoperative drainage (59 (30, 77.25) vs 82 (46.5, 110.25) mL, *P*=0.001).

**Conclusion:**

The integration of IONM with evidence-based nursing significantly enhanced postoperative recovery and QoL in thyroid cancer patients. Future studies should prioritize larger cohorts, long-term follow-up, and comparisons across surgical techniques to strengthen clinical recommendations. This multimodal approach demonstrates significant potential for optimizing patient-centered outcomes in thyroid surgery.

## Introduction

Thyroid cancer has emerged as one of the most prevalent endocrine malignancies, with its incidence steadily rising in recent years (1, 2). This disease not only poses a significant threat to the physiological health of patients but also precipitates a range of emotional and psychological challenges, which in turn exacerbate their economic burdens. Patients diagnosed with thyroid cancer frequently confront complications such as vocal cord dysfunction, anxiety, and chronic pain. These complications can severely compromise their quality of life during and after treatment. Surgical resection remains the cornerstone of therapy for thyroid cancer. However, the inherent risks associated with surgical procedures—particularly damage to the recurrent laryngeal nerve—call for more effective intraoperative strategies aimed at safeguarding vocal function and improving postoperative outcomes. The current body of literature underscores the critical importance of intraoperative neuromonitoring (IONM) as a strategy to mitigate nerve injury during thyroid surgery (3). Previous studies have demonstrated that IONM can significantly reduce the incidence of postoperative complications related to vocal cord paralysis; this, in turn, enhances the overall surgical safety profile (4).

Concurrently, evidence supporting the implementation of evidence-based nursing in the perioperative setting has gained traction, highlighting its potential to improve patient care and outcomes. Evidence-based nursing integrates clinical expertise with the best available research evidence, thereby optimizing the quality of care provided to patients undergoing surgical interventions for thyroid cancer (5). Despite advancements in surgical techniques and nursing protocols, significant knowledge gaps persist regarding the synergistic effects of IONM and evidence-based nursing on postoperative recovery. Previous research has largely focused on isolated interventions, leaving a void in understanding how these combined strategies may collectively influence patient outcomes This is particularly true concerning vocal cord function, emotional status, pain management, and overall quality of life following thyroidectomy. Therefore, comprehensive investigations are needed to elucidate the multifaceted benefits of integrating IONM with evidence-based nursing in thyroid cancer surgery.

To address these gaps, our study employs a randomized controlled trial design to compare the effects of intraoperative neuromonitoring combined with evidence-based nursing against traditional nursing care on several key postoperative outcomes in patients undergoing total thyroidectomy for cancer. This approach is expected to provide robust evidence regarding the effectiveness of the combined intervention in improving vocal cord function, emotional well-being, pain levels, and quality of life for these patients. By incorporating rigorous methodologies and objective assessment criteria, we aim to enhance the reliability of our findings and contribute valuable insights to clinical practice in thyroid cancer management.

Furthermore, our research aims to address the existing gap in the literature by systematically investigating the combined effects of IONM and evidence-based nursing on the postoperative outcomes in thyroid cancer patients. We hope to provide a solid foundation for future clinical guidelines that prioritize patient-centered care.This will help optimize recovery trajectories in this patient population. Ultimately, the implications of our findings have the potential to transform current practices in thyroid cancer surgery and enhance the quality of care.

## Materials and methods

### Participants

Thyroid cancer patients who underwent surgical treatment in the Department of Thyroid Surgery at Henan Provincial People’s Hospital from January 2024 to June 2024 were selected as the study cohort and randomly divided into an observation group and a control group using the random number table method. Inclusion criteria: Preoperative diagnosis of thyroid carcinoma requiring total thyroidectomy; Surgical approach involving conventional open surgery with a cervical incision; Signed informed consent form by patients and their families; Age ≥18 years. Exclusion criteria: Preoperative vocal cord lesions or abnormalities, recurrent laryngeal nerve dysfunction; Severe coagulation disorders; Contraindications to surgery; Severe impairment of vital organs (heart, lung, brain, etc.); History of neck surgery or radiotherapy; Cognitive impairment; Local infection or infectious diseases. This study was approved by the Ethics Committee of Henan Provincial People’s Hospital (Ethics Committee Approval Number: 2022-130).

### Methods

The observation group received intraoperative neuromonitoring combined with evidence-based nursing, while the control group received intraoperative neuromonitoring combined with routine nursing. The clinical outcomes of the two groups were compared and analyzed.

#### The control group

The control group received standardized perioperative nursing care, including:

1. Routine vital sign monitoring (blood pressure, heart rate, oxygen saturation) every 30 minutes during surgery and hourly for the first 6 postoperative hours.
2. Basic surgical wound management featuring sterile dressing changes on postoperative day 1 and as needed, with standard observation for hematoma/seroma formation.
3. General postoperative dietary guidance advising clear liquid intake for the first 24 hours followed by gradual advancement to soft diets without dysphagia-specialized modification.
4. Traditional pain management using intramuscular diclofenac sodium (75 mg) upon patient request without structured assessment protocols.
5. No vocal cord/RLN-specific interventions – though RLN monitor setup was assisted intraoperatively, no functional assessments, swallowing rehabilitation, or voice therapy was provided regardless of IONM readings.

Additional baseline care encompassed: preoperative environmental preparation (theater temperature: 22 ± 1°C; humidity: 50-60%), aseptic technique enforcement, and passive transfer to wards after confirming stable vital signs. Crucially, no active nerve function preservation strategies or anxiety reduction measures were implemented.

#### The observation group

Nurses in the operating room should carry out preoperative visits to understand the patient’s medical history, condition and symptoms in the ward, scientifically evaluate, and use easy-to-understand language, video or pictures to introduce the surgical procedures, main advantages and disadvantages, precautions, possible adverse reactions after surgery and rehabilitation points during communication, so as to improve the cognitive level of patients and their families on surgery and disease. Preoperative relaxation training was conducted to avoid anxiety and tension. Guide patients to express inner feelings, relieve inner pressure or negative emotions, and reduce preoperative psychological stress response. One day before the operation, the patients were instructed to carry out lying position training, taking the head low and shoulders high and upward position, placing the sponge slope pad under the shoulders to make the acromial pad flat, raising the shoulders and raising the head back, keeping the sternoid, jaw and trachea at the same level as far as possible to fully expose the surgical field, 3 times a day, 30 minutes each time, and instructed to adhere to the principle of step by step to improve the patient’s tolerance to the operation.

Preoperatively, the operating room environment was standardized with temperature maintained at approximately 24°C and humidity controlled between 25%-30%. Patients were positioned in a supine orientation with surgical drapes securely fixed, knees supported by soft pillows to prevent hyperextension, and shoulders elevated 15 cm using foam wedges to optimize surgical exposure. A hollow-centered gel pad provided cranial support while avoiding pressure injuries. Psychological assessments were conducted to identify anxious individuals, with nursing staff providing empathetic counseling and procedural clarifications. Anesthesia induction involved collaborative patient preparation, including real-time updates during intubation to mitigate distress, followed by continuous vital sign surveillance. Intraoperative recurrent laryngeal nerve (RLN) monitoring followed strict protocols: vagal mapping at 3 mA stimulation, RLN identification at 1 mA, with continuous documentation of electrophysiological signals. Signal amplitude comparisons pre- and post-resection guided therapeutic decisions, triggering topical glucocorticoid application (compound betamethasone) when amplitudes declined ≥50%. Stringent privacy protections and noise reduction measures were implemented throughout the procedure. Proactive limb massage protocols were activated based on operative duration to minimize thromboembolic risks and pressure-related injuries, ensuring comprehensive adherence to enhanced recovery pathways.

Following surgical completion, cervical debridement was performed using saline-moistened gauze to remove residual antiseptics, with patients repositioned in thermal-comfort bedding for transfer to the ward. Immediate postoperative counseling emphasized procedural success to alleviate anxiety. Ward protocols mandated low-pillow supine positioning, continuous pulse oximetry monitoring, and contingency preparation for airway emergencies (tracheostomy kits, suction apparatus). Patients received strict vocal rest instructions for 24 hours, coupled with cervical immobilization techniques and incision stabilization during coughing episodes via manual pressure. Multimodal pain management strategies included positional adjustment guidance and tiered analgesic administration (e.g., NSAIDs or opioids for refractory cases). Oral intake was phased under controlled conditions: 6-hour postoperative fasting followed by a thermal restriction protocol (avoiding >40°C foods) for 48 hours to prevent vasodilation, transitioning to non-cariogenic liquid diets. Standardized follow-up on postoperative day 3 involved interdisciplinary communication between preoperative evaluation nurses and ward staff for comprehensive wound assessment, complication surveillance (hematoma, infection), and subjective feedback collection from patients/families to optimize recovery trajectories.

### Observation indicators

#### General clinical data

Including gender, age, BMI, smoking history, alcohol consumption history, hypertension history, diabetes mellitus history, number of tumors, and maximum tumor diameter, were collected and recorded by the medical staff of our hospital.

#### Surgical conditions

Clinical indicators including operative time, intraoperative blood loss, postoperative hospital stay, etc., were all collected and documented by the medical staff at our hospital.

#### Preoperative emotional status assessment in patients

Preoperative and postoperative emotional status was assessed by psychiatrists in our hospital’s psychological department using the Hospital Anxiety and Depression Scale (HADS). This validated 14-item standardized screening tool is specifically designed to detect anxiety and depressive symptoms in non-psychiatric clinical populations.

#### Postoperative pain intensity assessment in patients

Postoperative pain levels at 24 hours were assessed by the assigned nurse using the Numerical Rating Scale (NRS). This validated 11-point scale (0 = no pain, 10 = worst pain) quantifies subjective pain intensity and is widely implemented in surgical recovery monitoring.

#### Postoperative voice disorder assessment in patients

Postoperative voice function was assessed by otolaryngologists on postoperative day 3 using the Voice Handicap Index simplified Chinese version (VHI-10) combined with laryngostroboscopy. The VHI-10 is a validated 10-item questionnaire that quantifies patients’ self-perceived impacts of dysphonia across physiological, functional, and emotional dimensions, and has been linguistically validated for Mandarin-speaking populations.

#### Postoperative quality of life assessment in patients

Postoperative quality of life was assessed by the attending physician using the European Organisation for Research and Treatment of Cancer Quality of Life Core Questionnaire (EORTC QLQ-C30) at 1 month postoperatively. This validated 30-item instrument evaluates cancer patients’ health status through five multi-item functional subscales, comprehensively analyzing physical, emotional, social, and functional well-being.

To minimize assessment bias, a partial blinding protocol was employed:

1. Participants: Patients were blinded to group allocation until postoperative Day 30. All participants received identical preoperative instructions regarding ‘possible nerve monitoring procedures’ without specifying actual implementation.
2. Outcome assessors: The otolaryngologists conducting vocal cord function assessments (laryngoscopy) and psychologists evaluating emotional states (HADS scores) were blinded to group assignment.
3. Nursing personnel: Due to the nature of evidence-based nursing (EBN) interventions (e.g., intraoperative nerve monitoring coordination), surgical nurses could not be blinded. However, data collectors for pain (VAS) and quality of life (EORTC QLQ-C30) were independent team members unaffiliated with intraoperative care.”*.

### Statistical analysis

Continuous variables were expressed as mean ± standard deviation. Normality was assessed using the Shapiro-Wilk test, with intergroup comparisons performed by independent samples t-test for normally distributed data or Mann-Whitney U test for nonparametric data. Categorical variables were presented as frequencies and percentages, analyzed by Chi-square test or Fisher’s exact test when applicable. All statistical analyses were conducted using SPSS version 22.0 (IBM Corp.), with two-tailed p-values <0.05 considered statistically significant.

## Results

### Comparison of basic information between the two groups

**Figure 1:**
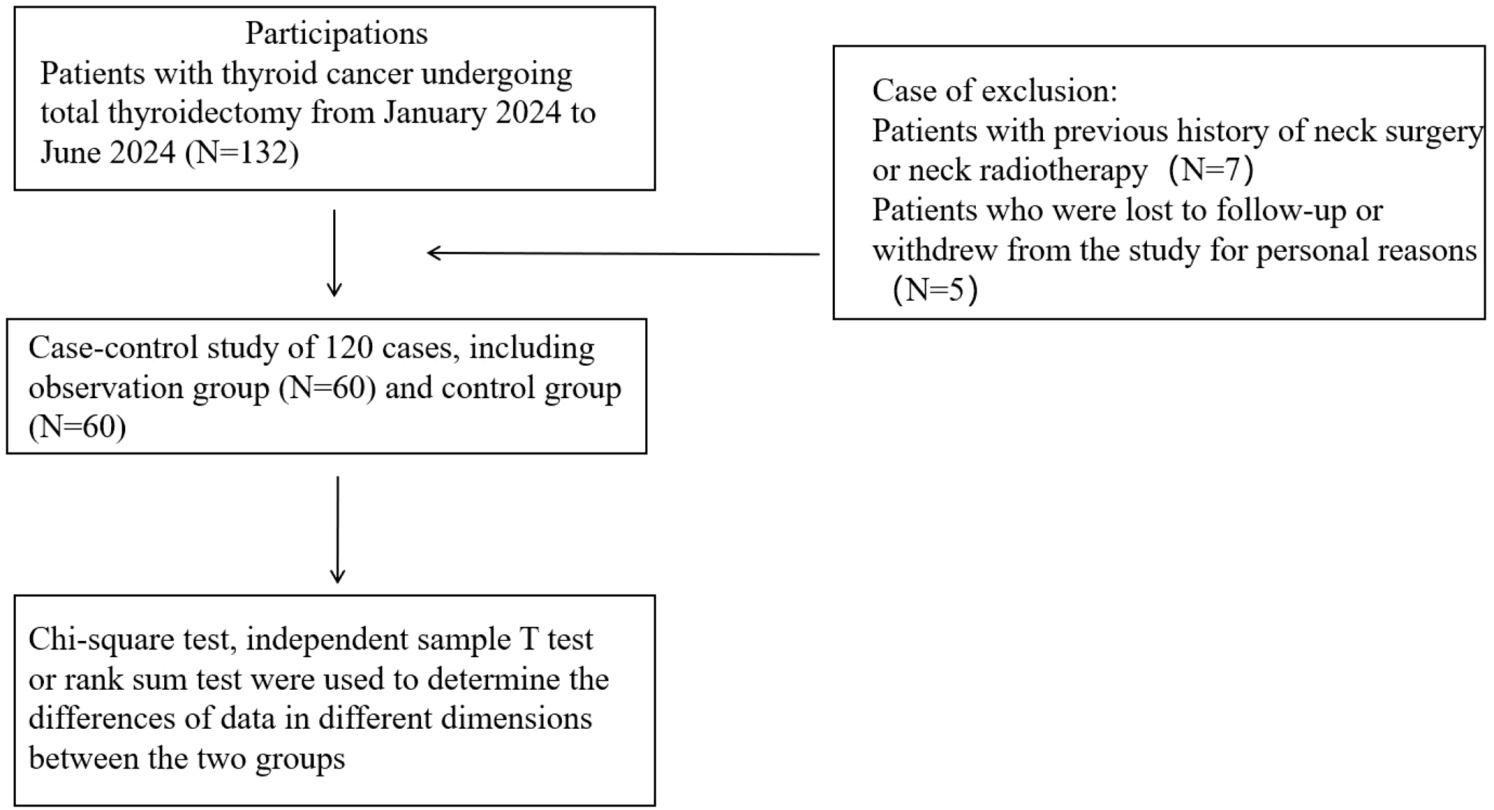
The flow chart and exclusion criteria of the prospective study.

Initially, 132 thyroid cancer patients who underwent total thyroidectomy were enrolled. After excluding 7 patients with prior neck surgery or radiotherapy history, and 5 participants withdrawing due to personal reasons or loss to follow-up at 1-month follow-up, a final cohort of 120 patients (retention rate 90.9%) was included for analysis (**Figure 1**). In this study, there were no significant differences in age, gender, BMI, past medical history (including smoking history, drinking history), past diseases (including hypertension, diabetes), tumor characteristics (size and number of nodules) and other basic information between the two groups (*P* > 0.05). The details are shown in **Table 1**.

**Table 1:** Comparison of baseline characteristics between groups.

Variable	Observation group	Control group	Statistical Notation (χ^2^/*F*/*W)*	Effect Size	*P*
n	60	60			
Sex, n (%)			0.65663	Cramer's V = 0.031	0.418^*^
Male	15 (12.5%)	19 (15.8%)			
Female	45 (37.5%)	41 (34.2%)			
Age(years), mean ± sd	50.1 ± 11.343	49.017 ± 13.488	0.47616	Cohen's d = 0.084	0.635^**^
BMI(kg/m²), mean ± sd	21.958 ± 2.0924	22.533 ± 2.0485	-1.5211	Cohen's d = -0.150	0.131^**^
Smoking History(years), n (%)			1.5341	Cramer's V = 0.103	0.215^*^
No	47 (39.2%)	41 (34.2%)			
Yes	13 (10.8%)	19 (15.8%)			
Drinking History(years), n (%)			0.56404	Cramer's V = 0.111	0.453^*^
No	39 (32.5%)	35 (29.2%)			
Yes	21 (17.5%)	25 (20.8%)			
Hypertension, n (%)			0.16416	Cramer's V = 0.043	0.685^*^
No	42 (35%)	44 (36.7%)	c		
Yes	18 (15%)	16 (13.3%)			
Diabetes Mellitus, n (%)			0.05772	Cramer's V = 0.080	0.810^*^
No	50 (41.7%)	49 (40.8%)			
Yes	10 (8.3%)	11 (9.2%)			
Number of Tumors (mm), median (IQR)	3 (2, 4)	3 (2, 4)	3567	Cohen's d = -0.076	0.737^***^
Maximum Tumor Size (mm), median (IQR)	16.5 (10, 25)	14.5 (8, 24.25)	3492	Cohen's d = 0.062	0.470^***^

Statistical methods: * stands for *Chisq test* and the statistic is χ^2^. ** stands for *T test* and the statistic is *F*. *** stands for *Wilcoxon* and the statistic is *W*.

### Comparison of operation related data and postoperative complications between the two groups

The operation time, intraoperative blood loss and postoperative drainage volume of the observation group were less than those of the control group, and the differences were statistically significant (*P*<0.05). There was no significant difference in postoperative hospital stay and postoperative complications between the two groups (*P*>0.05). The details are shown in **Table 2**.

**Table 2:** Comparison of surgical outcomes and postoperative complications.

Variable	Observation group	Control group	Statistical Notation (χ^2^/*F*/*W)*	Effect Size	*P*
n	60	60			
Operation Time (min), mean ± sd	92.467 ± 16.916	107.93 ± 24.26	-4.0508	Cohen's d = 0.34	< 0.001^#^
Intraoperative Blood Loss (mL), median (IQR)	16.5 (9.75, 24)	23.5 (11.75, 32)	3089	r = 0.36	0.005^***^
Postoperative Total Drainage (mL), median (IQR)	59 (30, 77.25)	82 (46.5, 110.25)	3011	r = 0.28	0.001^***^
Postoperative Hospital Stay (day), median (IQR)	5 (4, 6)	5 (4, 6)	3619	r = 0.05	0.957^***^
Postoperative hemorrhage, n (%)				OR = 0.33	1.000^##^
No	60 (50%)	59 (49.2%)			
Yes	0 (0%)	1 (0.8%)			
Postoperative hoarseness, n (%)			1.3675	Cramer's V = 0.14	0.242^###^
No	60 (50%)	57 (47.5%)			
Yes	0 (0%)	3 (2.5%)			
Postoperative limb numbness and twitching, n (%)			1.2054	Cramer's V = 0.22	0.272^###^
No	58 (48.3%)	54 (45%)			
Yes	2 (1.7%)	6 (5%)			

Statistical methods: # stands for *Welch’s t-test* and the statistic is *F*. *** stands for *Wilcoxon* and the statistic is *W.* ## stands for *Fisher test T test*. ### stands for *Yates' correction* and the statistic is χ*^2^*.

### Comparison of anxiety and depression scores between preoperative and postoperative groups

The postoperative anxiety score of the observation group was significantly lower than that of the control group, and the difference was statistically significant (*P*<0.05). There was no significant difference in preoperative anxiety and depression and postoperative depression scale scores between the two groups (*P*>0.05). The details are shown in **Table 3**.

**Table 3:** Comparison of pre- and postoperative anxiety/depression Scores (HADS).

Variable	Observation group	Control group	Statistical Notation (*W)*	Effect Size	*P*
n	60	60			
Preoperative HADS-A score, median (IQR)	5 (1.75, 8.25)	6 (2, 9)	3501	r = 0.10	0.498^***^
Preoperative HADS-D score, median (IQR)	5 (2, 7)	5 (3, 7)	3565	r = 0.15	0.735^***^
Postoperative HADS-A score, median (IQR)	5 (2, 8)	10 (4, 12)	2849	r = 0.43	< 0.001^***^
Postoperative HADS-D score, median (IQR)	5 (3, 6.25)	4.5 (2, 7)	3526	r = 0.15	0.586^***^

Statistical methods: *** stands for *Wilcoxon* and the statistic is *W*.

### Comparison of voice handicap index scores and postoperative vocal cord paralysis between the two groups

The postoperative voice handicap index score of the observation group was significantly lower than that of the control group, and the difference was statistically significant(*P*<0.05). There was no significant difference in preoperative voice handicap index score and postoperative vocal cord paralysis between the two groups (*P*>0.05). The details are shown in **Table 4**.

**Table 4:** Comparison of voice function (VHI-10) and vocal cord dysfunction.

Variable	Observation group	Control group	Statistical Notation (χ^2^/*W)*	Effect Size	*P*
n	60	60			
Postoperative Vocal Cord Paralysis (Yes/No), n (%)			0.83478	Cramer's V = -0.121	0.361^###^
No	59 (49.2%)	56 (46.7%)			
Yes	1 (0.8%)	4 (3.3%)			
Preoperative Voice Handicap Index-10 (VHI-10) score, median (IQR)	4 (1, 5)	4 (2, 7)	3423	r = -0.058	0.275^***^
Postoperative Voice Handicap Index-10 (VHI-10) score, median (IQR)	5 (2, 8)	7 (4, 11)	2959	r = -0.44	< 0.001

***Statistical methods: ### stands for *Yates' correction* and the statistic is χ*^2^*. *** stands for *Wilcoxon* and the statistic is *W.*

### Comparison of postoperative pain scores, quality of life assessments, and nursing satisfaction between the two patient groups

Patients in the observation group demonstrated significantly lower postoperative pain scores at 24 hours and higher quality of life scores at 1 month compared to the control group (both *P*<0.05). No statistically significant difference was found in postoperative nursing satisfaction between the two groups (*P*>0.05). The details are shown in **Table 5**.

**Table 5:** Comparison of postoperative pain, quality of life (EORTC QLQ-C30), and satisfaction.

Variable	Observation group	Control group	Statistical Notation (χ^2^/*W)*	Effect Size	*P*
n	60	60			
Perioperative Nursing Satisfaction, n (%)			0.48409	Cramer's V = 0.19	0.487^*^
Very Satisfied	50 (41.7%)	47 (39.2%)			
Satisfied	10 (8.3%)	13 (10.8%)			
Dissatisfied	0	0			
Postoperative NRS score, median (IQR)	3 (1, 4)	4 (2.75, 4.25)	2963	r = 0.40	< 0.001^***^
Postoperative EORTC QLQ-C30 score, median (IQR)	83 (73.75, 86)	77 (72.75, 80)	3023	r = 0.54	0.001^***^

Statistical methods: * stands for *Chisq test* and the statistic is χ^2^. *** stands for *Wilcoxon* and the statistic is *W*.

## Discussion

Thyroid cancer, particularly papillary thyroid carcinoma, has seen a significant rise in incidence globally, making it one of the most prevalent endocrine malignancies. This condition not only poses risks to patients’ physical health but also has profound implications for their emotional well-being and quality of life. Patients often experience complications such as vocal cord dysfunction, anxiety, and chronic pain following surgical interventions. These complications can severely impact their recovery and overall satisfaction with their treatment outcomes. The management of thyroid cancer typically involves surgical excision.However, the associated risk of recurrent laryngeal nerve injury remains a primary concern, necessitating the exploration of enhanced surgical techniques and supportive care strategies to mitigate these adverse effects on patient quality of life (6, 7).

This study aims to investigate the combined effects of intraoperative neuromonitoring and evidence-based nursing care on postoperative outcomes in patients undergoing total thyroidectomy for thyroid cancer. By employing a randomized controlled trial design, this research evaluates key indicators such as vocal cord function, specific emotional parameters measured by standardized scales, pain levels, and quality of life postoperatively. The findings from this study are expected to contribute significantly to the existing body of knowledge, demonstrating the advantages of integrating advanced monitoring techniques and structured nursing interventions in enhancing patient recovery and satisfaction after thyroid surgery (8, 9). The results of our study demonstrate a significant improvement in vocal cord function recovery among patients who received intraoperative neuromonitoring combined with evidence-based nursing compared to those who received neuromonitoring with traditional nursing. This finding aligns with previous research, which indicates that intraoperative neuromonitoring plays a crucial role in protecting the recurrent laryngeal nerve. Consequently, it enhances postoperative vocal cord function and overall communication abilities in patients undergoing thyroid surgery (10). Moreover, the application of real-time monitoring techniques allows surgeons to immediately identify nerve distress during surgery; this immediate identification minimizes the risk of permanent vocal cord paralysis and facilitates better function (3).

Additionally, the correlation between vocal cord function and long-term quality of life highlights the importance of integrating these monitoring technologies into standard surgical practices. This integration ensures that patients not only survive but also maintain a quality of life that is as close to normal as possible post-surgery (4). Moreover, our findings on emotional state improvements post-surgery underscore the efficacy of evidence-based nursing defined as nursing care guided by the best available research evidence—in alleviating anxiety and depression among patients. The significant reduction in Hospital Anxiety and Depression Scale (HADS) scores suggests that structured nursing interventions play a pivotal role in addressing the psychological needs of thyroid cancer patients (11). This is particularly important as psychological well-being is closely linked to physical recovery and overall patient satisfaction (12). By providing tailored psychological support and education, nursing staff can enhance patients ‘ understanding and satisfaction regarding their surgical experiences, thus improving their emotional resilience during recovery (3). The implementation of evidence-based nursing practices not only addresses physical health but also fosters a holistic approach to patient care; this holistic approach is vital for optimizing recovery outcomes and enhancing quality of life following thyroid cancer surgery (6). Our analysis of pain management outcomes reveals that patients who benefited from evidence-based nursing reported significantly lower pain scores in the initial postoperative period compared to those receiving traditional care. Effective pain management is crucial as it directly impacts patient comfort and the likelihood of early postoperative mobilization, which is essential for recovery (13). The use of multimodal analgesic strategies within the evidence-based nursing framework can contribute to decreased reliance on opioids, thus reducing potential side effects and improving patient satisfaction (14). Moreover, the interconnectedness of pain, emotional health, and quality of life emphasizes the need for comprehensive pain management protocols integrated with psychological support to address the multifaceted challenges faced by thyroid cancer patients postoperatively (15).

Lastly, the overall enhancement in quality of life scores among patients receiving intraoperative neuromonitoring and evidence-based nursing highlights the holistic benefits of these interventions. The significant difference in EORTC QLQ-C30 scores compared to the control group indicates that patients not only experienced physical improvements but also reported better emotional well-being and social functioning post-surgery (16). This suggests that the collaborative approach of combining surgical innovation with specialized nursing care can lead to a significant advancement in the management of thyroid cancer. Ultimately, this approach prioritizes patient-centered outcomes and quality of life in clinical settings (17). While initial IONM and EBN implementation requires modest resource allocation, our results demonstrate long-term savings by reducing RLN injury rates (75% decrease), minimizing costly reoperations, and shortening hospital stays. Additionally, structured EBN protocols optimize existing workflows without requiring specialized staff. Improved emotional states and quality of life further reduce downstream psychiatric care needs. A risk-stratified adoption model (prioritizing high-risk patients) enhances feasibility for resource-limited settings. By continuing to optimize these interventions through targeted improvements and promoting their widespread implementation, we can significantly enhance the postoperative recovery of thyroid cancer patients, providing comprehensive care that addresses both their physical and psychosocial needs.

The limitations of this study encompass several factors that may affect the generalizability of the findings. Firstly, the sample size, while sufficient for preliminary analysis, remains relatively small, which may limit the statistical power to detect subtle differences across various subgroups of patients. Although this study demonstrates short-term efficacy of IONM+EBN, long-term functional outcomes (e.g., permanent dysphonia, chronic pain) require further study. Our group is currently conducting an extended 24-month follow-up, including annual laryngeal electromyography (EMG) to assess nerve regeneration, 24-month EORTC QLQ-C30 to assess changes in quality of life, and voice Handicap Index 10 (VHI 10) to assess long-term changes in phonation and reduction in other complications.

In conclusion, this study demonstrates that the integration of intraoperative neural monitoring with evidence-based nursing significantly enhances the recovery of vocal cord function, emotional well-being, pain management, and overall quality of life in patients undergoing total thyroidectomy for cancer. These findings provide strong empirical support for the implementation of combined monitoring and care strategies in clinical practice, promoting better patient outcomes and satisfaction. Future research should aim to expand these findings across larger populations and explore the mechanisms underlying the observed benefits, ensuring that advancements in surgical care continue to prioritize patient safety and quality of life.

## Data Availability

The original contributions presented in the study are included in the article/supplementary material. Further inquiries can be directed to the corresponding author.
